# eHealth Literacy: From Theory to Clinical Application for Digital Health Improvement. Results from the ACCESS Training Experience

**DOI:** 10.3390/ijerph182211800

**Published:** 2021-11-10

**Authors:** Roberta Bevilacqua, Stefano Strano, Mirko Di Rosa, Cinzia Giammarchi, Katerina Katka Cerna, Claudia Mueller, Elvira Maranesi

**Affiliations:** 1 Scientific Direction, IRCCS INRCA, 60124 Ancona, Italy; r.bevilacqua@inrca.it (R.B.); s.strano@inrca.it (S.S.); c.giammarchi@inrca.it (C.G.); e.maranesi@inrca.it (E.M.); 2 Unit of Geriatric Pharmacoepidemiology, IRCCS INRCA, 60127 Ancona, Italy; 3 Information Systems/IT for the Ageing Society, University of Siegen, 57076 Siegen, Germany; katerina.cerna@uni-siegen.de (K.K.C.); claudia.mueller@uni-siegen.de (C.M.)

**Keywords:** eHealth literacy, lifelong learning, training, older adults, digital inclusion

## Abstract

Skills, knowledge, and awareness of digital and technological tools are essential to improve the state of well-being and health of older adults and also to mitigate the condition of social isolation in the aging process. For this reason, it is necessary to implement a social learning of electronic/digital tools for health of older people to support the achievement of eHealth and digital competences. The paper reports the results of an Italian innovative eHealth training for the European project ACCESS. The training has been based on blended didactical and interactive educational techniques, aimed at collecting as many points of view as possible from older adults. A total of 58 older adults were recruited to attend a four-week training program, which included five modules. The results showed a statistical significant difference between the eHealth Literacy Scale (eHEALS) mean value before and after the course. A significant negative correlation was found between eHEALS and positive/total Survey of Technology Use (SOTU), suggesting an inverse relationship between positive/total SOTU and eHEALS. There is a strong positive and statistically significant relationship between satisfaction with the training and eHEALS. The results indicate that the intervention increased the digital competences of participants connected to health.

## 1. Introduction

Skills, knowledge, and awareness of digital and technological tools are essential to improve the state of well-being and health of older adults [1,2] and also to mitigate the condition of social isolation in the aging process, which has become even more serious since the beginning of the COVID-19 era [3,4]. 

In fact, social distancing measures forces individuals to minimize physical interactions that result in increasingly widespread and grave social isolation and loneliness. Health technologies can support older adults to mitigate the overwhelming impact of the pandemic situation and enhance well-being, while COVID-19 is an “alarm bell”, reminding that an immediate attention should be paid to improve eHealth literacy among older people to use technological/digital innovations as a “complementary tool” to provide care services [5]. During these contingencies, older adults are pushed to improve their technology skills and gained experience using online platforms, with a stronger motivation and a more favorable attitude toward digital or technological tools and learning opportunities, as a real necessity of daily life, or a ‘sink or swim’ moment for many reluctant or unskilled users [6]. Social isolation and loneliness are serious risk factors that can worsen older adults’ health status, and there is a need to realize innovative technology based interventions able to include the older person in the digital world, and mobilizing the resources more capable to support in daily life, as family members, or community-based networks [7]. Digital tools potentially offer a consistent health improvement, especially apps, as the most accessible and cost-effective solution, being easily ready-to-use on already widely accepted and purchased devices, even by older adults, such as smartphones or tablets, if older users are first provided with appropriate training [8].

Nevertheless, there are a plethora of ageing-specific barriers that may represent obstacles to the positive use of technological tools and the enhancement of eHealth literacy, such as multimorbidity and the presence of physical impairments or cognitive deterioration. Other major impediments concern financial resources (i.e., inadequate funding or reimbursement schemes); others are technical, as the compatibility between different eHealth tools or insufficient ICT support and infrastructures; or legal, as the absence of a legislative framework more clearly defined to support eHealth literacy achievement [9]. Similar barriers, listed in order of frequency, are also ranking in a recent systematic review focusing on supporting the use of telemedicine and eHealth tools. As “organizational barriers”, the first relates to cost and reimbursement (13%) and the second to aspects as legal liability, privacy, and confidentiality concerns and security of data (11%). On the other side, from the perspective of “patient barriers” that affect older adults, the age of the patient and level of education are the most blocking (17%); while inadequate eHealth or computer literacy is a significant impediment, summed with other adverse factors as bandwidth of dwelling, and state of unawareness of the existence of telemedicine products and services (14%) [10].

Similar argumentations suggest that it is mandatory to implement a social learning of electronic/digital tools for health of older people to support the achievement of eHealth and digital competences. 

eHealth literacy has been conceptualized as “the ability to seek, find, understand, and appraise health information from electronic sources and apply the knowledge gained to addressing or solving a health problem”, representing a factor of critical importance to counteract multiple access barriers to technology for older adults, related to motivation, material, skills, and usage spheres [11,12]. 

Training focused on eHealth literacy is becoming increasingly essential in the context of an ageing Europe: with the potential offered by digital tools, eHealth literacy can strengthen health literacy itself and enable a deeper knowledge and better self-management for patients, particularly for older adults [13,14]. 

In fact, eHealth training can directly improve motivation, reducing negative emotional factors such as stress, fear, and anxiety that block the use of eHealth technologies [15,16], as well as enhancing the degree of skills and ability to research, understand, and use digital health information, tools, or apps for self-management and well-being [17]. 

In the current state of the art, there is still a scarcity of theory-based learning interventions focused on eHealth literacy that assess the direct impact on health outcomes, and there is a need to develop and implement a high-quality research study design for this purpose [18]. 

Taking into account the framework of barriers and benefits, the paper reports the results of an innovative eHealth training, piloted in Italy during November 2020, involving 58 older people, for the European project ACCESS (MYBL-2016-3; Grant Agreement nr. 643850), aimed at (a) exploring, implementing, and evaluating new modes of socially embedded learning opportunities for older adults with low technical skills; (b) identifying ways to improve digital literacy in regard of internet skills and the everyday usage of assistive technologies in older individuals; (c) fostering a new learning culture for later-life learning.

The ACCESS eHealth training has the ambitious purpose of advancing toward a clearer definition and measurement of eHealth literacy in practical scenarios, improving the overall eHealth literacy and thus supporting the positive use of technology [19].

## 2. Materials and Methods

### 2.1. Description of the Training

The training was based on blended didactical and interactive educational techniques, aimed at collecting as many points of view as possible from older adults. The training’s program included 5 modules and was conducted over 4 weeks. On average, the duration of each session was 90 min. The sessions were conducted using the GoToMeeting platform. 

The overall training objectives refer to the three learning competencies of the European Qualifications Framework (EQF) reported in Table 1:

The training is divided on the following modules:

- Module I: Raising awareness of eHealth and health literacy: introduction to the aims and context of the ACCESS project; Achieving new knowledge: introduction to Health and eHealth literacy;
- Module II: Practicing new skills: digital health apps and skills with practice session;
- Module III: Practicing new skills: usability with practice session;
- Module IV: Social communication: inter-generational mode;
- Module V: Self-evaluation and sustainability of the improvement (final questionnaire).

*Module 1* introduced the objectives of the ACCESS project and analyses the definition and meaning of eHealth literacy. An overview of the initiatives and projects for the digitization of the older people in Italy is provided, with a focus on the barriers to the use of technological and digital health (eHealth) solutions (e.g., legal framework, private and sensitive data, limited cost-effectiveness of eHealth technology projects, multi-morbidity). The first part of the module is managed as a frontal lesson. In the second part, a discussion is conducted, in focus group mode, to reflect and share the point of view on approach and perceived images of technology. The aim is also to lead older participants to reflect on the concept acceptance/rejection of technological tools, trying to distinguish the barriers to access (motivational, digital skills, material, use) that are experienced more in everyday life. 

*Module 2* combines theoretical and practical aspects, guiding participants to know what health literacy and eHealth literacy is and providing information to improve health/eHealth literacy, in order to enhance knowledge and awareness of digital tools and applications more functional to psychophysical well-being. The second part of the lesson is managed as a practical exercise, starting from the selection of freeware digital health apps, to promote in older people a more autonomous and conscious management of health and increase the degree of health and digital literacy (eHealth). The objective is to provide participants with a selection of apps with health information from secure and verified official health systems’ sources, easy to use and usable even after the end of the training, so that they will be more familiar with, and gain greater utility from, the use of such tools in their daily lives.

*Module 3* is focused on the concept of usability of digital and technological services and devices, presented in frontal lesson mode. One of the theoretical assumptions to be learned by older adults is based on the idea that no technology is positive or negative in itself, but often the poor usability of tools causes frustration in use and consequent refusal. The scope is to acquire greater awareness of the problems or errors in use, trying to distinguish situations in which the ineffective use, or non-use, is due to poor user skills or, conversely, to an inadequate degree of usability of the service/technological tool. The second part of the module is conceived as a practical exercise, based on an example test of usability of online portals that offer a service of wide use, especially for the health of older people. This test, for instance, concerns the online booking center of medical examinations. In particular, the main usability problems of an online site, which can cause difficulties of use for the older population, are shown. 

*Module 4* is designed considering an intergenerational learning context: in agreement with a high school, students, coordinated by their teacher, conduct a lesson on the use of social networks (such as Instagram, Tik Tok, YouTube, Snapchat, etc.) to promote the use and social inclusion of the older adults. The direct involvement of so-called “digital natives” represents a great added value for older people, recalling the experiential/emotional interaction between grandparents and grandchildren. By describing the characteristics and functioning of some of the main web tools, apps, and socials in common use, the young students stimulate the older adults to think about current issues concerning the relationship with technology and the potential inherent in new forms of communication and sociality. This results in a reflection from the different point of view between a technological generation and a non-technological one.

*Module 5* aimed to assess the self-evaluation of enhanced awareness, knowledge, and skill levels of technological use and digital health literacy, with a concluding section regarding the evaluation of the training activity and teaching carried out. In the final session, the trainer describes to the participants the characteristics of the final questionnaire to support them in filling it out.

Moreover, the willingness to pay will be analyzed at the end of the training. In addition, an attempt will be made to analyze the relationship between familiarity with technology and appreciation of the intervention with the health literacy.

### 2.2. Subjects

The training is designed for older adults, sorted into groups of approximately 20 participants for online mode, led by a trained facilitator. For the pilot, it was possible to recruit 58 older adults from all provinces of the territory, with a mean age of 68.2 (±5.0) years, in agreement with a regional union of retirees (FNP CISL Marche). For a better management and a more effective learning, the participants were subdivided into three distinct groups, independently from education level and digital skills. The recruitment of the participants was conducted during the COVID-19 lockdown, through the NGO’s contacts. An email was sent to the newsletter and the interested persons contacted the researcher directly. As a prerequisite to take part in the study, the availability of an internet connection and a pc or tablet were required, in order to be able to follow the group lessons. The materials were sent to the participant after each lesson by email.

### 2.3. Procedure

Before starting the training, informed consent was provided to the participants and a copy of the signed informed consent was collected by post. During the first session, a questionnaire was presented to the participants containing information on experience with technology, eHealth literacy, socio-demographic information.

The preliminary questionnaire was composed by:

- an ad hoc checklist to collect socio-demographic information;
- the Survey of Technology Use (SOTU) from the Matching Person and Technology Model. SOTU is composed by a 29-item checklist which inquires into current experiences and feelings toward technologies. All items are presented in a three-point semantic differential format to elicit the consumer’s feelings towards these influences (e.g., positive, neutral, negative); in this way, three subscales are scored, highlighting positive, negative, or neutral past experiences with technology as well as a total score [20].
- The Italian version of the eHealth Literacy Scale (eHEALS), an 8-item measure of eHealth literacy developed to assess consumers’ combined knowledge, comfort, and perceived skills at finding, evaluating, and applying electronic health information to health problems [21].

During the last session, a final questionnaire was administrated including:

- the eHealth Literacy Scale (eHEALS) [22];
- an ad hoc checklist, consisting of 8 questions to be rated on a 5-point Likert scale, to assess the satisfaction with the training, from 1 “Do not agree at all” to 5 “Completely agree”;
- one ad hoc question to assess the availability of pay for the course.

### 2.4. Statistical Analysis

Continuous variables were reported as either mean and standard deviation (SD) or median and interquartile range (IQR) on the basis of their distribution (assessed using the Shapiro–Wilk test). Comparison of variables between groups were performed by an unpaired Student *t*-test or Mann–Whitney U test, as appropriate. Categorical variables were expressed as the absolute number and percentage and statistical significance was assessed by Pearson’s Chi-square test. In a second step, the analysis of follow-up data was carried out to evaluate the effectiveness of the intervention. Frequencies of eHEALS items were compared between baseline and follow-up via Pearson’s Chi-square test. The overall mean values of the eHEALS scale were compared between the two time points and statistical significance was assessed with a paired *t*-test. Willingness to pay was analyzed reporting the mean and SD of the program satisfaction for each cost item: statistical significance was assessed by one-way analysis of variance (ANOVA). The relationship between SOTU subscales, overall SOTU, satisfaction with the training program, and eHEALS was estimated by calculating the pairwise correlation coefficients. The statistical significance was set at *p* < 0.05. Statistical analysis was conducted using the Stata 15.1 Software Package for Windows (StataCorp, College Station, TX, USA).

## 3. Results

The demographic, social, and eHealth literacy characteristics of the sample are reported in Table 2. No statistically significant difference emerged between age groups.

Table 3 shows the eHealth literacy value at the baseline (T0, first session) and at the end of the course (T1, last session). The value ranges from 1 (in completely disagreement) to 5 (completely agree). Frequencies for each item of the scale improved significantly from baseline to follow-up. A statistically significant difference was also found between the overall eHEALS mean value before (24.3 ± 8.9) and after (28.4 ± 8.1) the course (*p* = 0.001).

Another interesting aspect is the response of the sample to the question of how much they would pay for the course. In fact, 18 people would pay more than 70 € (Table 4). There is also a statistically significant relationship of the willingness to pay and the mean satisfaction of the course (people who were willing to pay more were also more satisfied about the course).

Table 5 shows the correlation coefficient between the eHEALS scale at the end of the course and SOTU, together with the significance of this relationship. The negative correlation between eHEALS and positive SOTU and total SOTU are significant (*p* = 0.048 and *p* = 0.032), suggesting an inverse relationship between positive/total SOTU and eHEALS. There is a strong (almost 80%) positive and statistically significant relationship between satisfaction with the training and eHEALS.

## 4. Discussion

The eHealth concept can be a starting point to better understand how to support older people in using devices to take care of their own health. The absence of standardized eHealth and technological training for older people is a matter of particular relevance for the support of health and well-being during the ageing process. In the current state of the art, in fact, the weak link is represented by the lack of a training that is not limited to teaching how to use PCs, tablets, or smartphones as a more or less advanced practical skill, but that includes broader concepts of paramount importance for the construction of realistic expectations towards technology, such as usability, i.e., how to avoid frustration in use when a usability problem is recognized; a user-centered design and participatory approach, i.e., feeling be part of the process of designing technology and not feeling excluded; and the awareness of access barriers to technology. 

The most important outcome of the ACCESS training is the decisive improvement of the eHealth literacy itself: the intervention has demonstrated to significantly improve the digital health competences of the older participants, especially the ability to retrieve useful and reliable health information on the Internet to increase the ability of older adults to use Internet links and find desired health-related information, as already demonstrated by a study in the field [23]. This main finding is in line with the literature, which highlights how eHealth literacy interventions may be effective in diminishing anxiety during technology-related activities: more experience and expertise of technology result in reduced computer stress and higher levels of eHealth literacy, but it is nonetheless necessary “to identify educational interventions” to support older people to successfully use technology and enhance eHealth literacy [24]. For this reason, it can be supposed that reduced fear and increased acceptance of technology, raised awareness and confidence as well as self-management of health are success factors, achievable with innovative eHealth training such as the one proposed, even if more extensive studies are needed, including evidence-based trials. However, it seems that, especially in the short term, the ACCESS training provided a responsive and constructive action to the emergency of digital exclusion for older people to the eHealth domain, improving the sense of socialization and being together, sharing and connecting with others and increasing the awareness of the potential of digital tools for health. Moreover, the positive evaluation of the training at the end of the pilot suggests the probability of sustaining the improvement of new skills and knowledge acquired by older adults. Despite this, the absence of a follow-up evaluation after some months by the end of the pilot represents a limitation of the present study. 

From a very practical point of view, the participants report to like the format of the training, consistent with the literature on e-learning programs on eHealth literacy [25]. In fact, all the participants completed the training and showed high adherence to sessions and practical activities. The duration of each session was adequate; longer sessions would have been too tiring for the older people. Additionally, the duration of the training was felt as adequate, allowing the participants to repeat the exercises sufficiently and discuss the proposed topics deeply.

Moreover, the results reported in Table 4 suggest that the participants that had less positive experiences with technology (SOTU-positive) or, more in general, had less past experience with technology (SOTU total) perceived a higher improvement of their eHealth literacy after the course, highlighting the positive role of group training such as ACCESS, to support the hard-to-reach group of older people, often excluded by the opportunities of being familiar with technological devices. 

A recent analysis of the literature [18] has examined 23 studies, aimed at assessing the impact of different eHealth or health literacy trainings. Of these, only 8 studies have targeted eHealth literacy as a main outcome variable, but none of them have examined the relationship with a specific health outcome. Moreover, six of them have used instructional materials developed by the National Institute on Aging (NIA) of the National Institutes of Health (NIH). The authors recommended to deeply analyze the relationship between eHealth literacy and specific health outcomes, especially to be conducted in clinical settings, where participants may experience less difficulty in reporting health or health literacy problems. Despite those limitations, which are valid also for our case, the ACCESS training seems to differ from the others: while all the reported studies are highly focused on specific health topics, our training was designed to create an overall perspective towards eHealth in the participants; in other terms, it is not linked to a specific device use or health problems, but it introduces concepts such as usability, awareness, and knowledge on eHealth and health literacy, with the aim of creating a positive representation of technologies, not dependent on the specific problem/device. This finding suggests the importance of providing older adults with a variety of offers that better match the heterogeneous needs of the group. Such heterogeneous needs have been documented when it comes to the need for technology overview [26]. By enabling the older participants to apply the different technologies in the health area, we create the possibility for the older adults to create solutions to their own individual health problems. 

In this way, it seems essential to develop a specific theoretical framework to guide eHealth intervention research with older adults as well as to improve eHealth interventions in real settings. In our case, in addition to the well-known definition of health and eHealth literacy, we have started our assumption from the overall concept of technology literacy, in its definition from ITEA (2000/2002) that refers to “one’s ability to use, manage, evaluate, and understand technology. In order to be a technologically literate citizen, a person should understand what technology is, how it works, how it shapes society and in turn how society shapes it”. If we try to redefine the concept of eHealth literacy by introducing the wider perspective adopted by technology literacy, it is possible to define an “e-health technologically literate person as is comfortable with and objective about the use of technology for health, by involving a vision where every person has a degree of knowledge about the nature, behavior, power and consequences of many aspects of technology—especially for health—from a real world perspective” (adapted by ITEA). 

The empowerment of older people’s eHealth literacy is of crucial importance, especially to navigate in the COVID-19 infodemic; for this purpose, researchers, academics, and health authorities have to consider eHealth literacy as a crucial factor to facilitate infection prevention, for example, [27]. Recent findings, in fact, suggest that health care professionals often overestimated the capability of the citizens to handle health-related information, or in other terms, their eHealth or Digital Health competences [28]. As shown in recent evidence, health professionals have a key role in vehiculating health literacy [29,30].

Moreover, together with family, health professionals are important mediators of technology use for health. For this reason, a dedicated and complementary training on how to support older people with low eHealth competences should be developed also for health professionals, in order to provide them practical strategies for saving time in habitual activities, for example, and reducing the burden of care. 

During the ACCESS study, the online modality was appreciated by the participants and allowed the management of the course during the lockdown due to COVID-19, but the provision of digital health courses for older adults could be part of a long-term inclusive local digitalization strategy [31,32] to counteract the exclusion from digital health services of people with limited health literacy and digital skills, who are disproportionally represented in disadvantaged and ‘hard-to-reach’ groups [33]. 

To summarize, it is possible to recognize the positive effect of the ACCESS training on eHealth literacy, highlighting the potential of educational intervention in providing useful knowledge and skills to overcome erroneous representations of technology as well as misuse of the health technological devices. The expected benefit of training as ACCESS is the inclusion of older people in the technological world, on a global perspective, as well as the improvement of perceived self-efficacy in dealing with technology and health, with positive pitfalls on the health status. Despite its undeniable wealth, there are some limitations that need to be taken into account to improve the study design of the training. First of all, future pilots should take into consideration the opportunity of including a control group as well as a larger sample to assess the efficacy of the training from a more evidence-based perspective. Moreover, follow-up evaluations should be performed in order to assess the maintenance of the improvement after the training. Future plans should also include sustainability strategies, as the educational training should be integrated not only in the social and community services but should also be delivered by public and private health providers.

## 5. Conclusions

To conclude, fostering eHealth literacy in older adults hosts the potential to improve the wellbeing of older adults in today’s rapidly developing society. Currently, suitable training programs targeting older adults combining health and digital literacy are missing. In our paper, we introduce an eHealth training program, which was delivered to older adults across four weeks through online activities. Our results indicate that the intervention increased the digital competences of participants connected to health. In order to effectively address eHealth literacy, a comprehensive approach is required as future work, building on innovative evidence-based tools and best practices, such as the proposed training course, to simultaneously reach out and target citizens, communities, professionals, the industry, and health and social care systems, with specific attention paid to the inclusion of ‘hard-to-reach’ people so as to ensure that no one is left behind.

## Figures and Tables

**Table 1:** The three learning competencies of the European Qualifications Framework (EQF).

Competencies	Training Objectives
Awareness and critical knowledge of health and eHealth literacy	To inform and educate—Know about health and eHealth literacy issues, their impact and interventions to tackle health and eHealth literacy problems
Advanced skills in relation to health and eHealth literacy older adults interactions with digital tools	To teach skills—Develop older-adult-centered skills to address problems with health and eHealth literacy
Sustainability of skills developed and application in practice.	To support behavior change—Adopt, change, and maintain behavior to address health and eHealth literacy problems

**Table 2:** Demographic, social, and eHealth literacy characteristics of the sample.

Variable	Overall n = 58	Age 50–69 n = 31	Age 70+ n = 27	*p*
Gender, n (%)				0.531
Male	34 (58.6%)	17 (54.8%)	17 (63.0%)	
Female	24 (41.4%)	14 (45.2%)	10 (37.0%)	
Marital status, n (%)				0.555
Married (cohabiting with husband/wife)	43 (74.1%)	22 (71.0%)	21 (77.8%)	
Separated, Divorced, Single, Widowed	15 (25.9%)	9 (29.0%)	6 (22.2%)	
Education level, n (%)				0.459
Primary education	5 (8.6%)	4 (12.9%)	1 (3.7%)	
Secondary education	41 (70.7%)	21 (67.7%)	20 (74.1%)	
Tertiary education	12 (20.7%)	6 (19.4%)	6 (22.2%)	
Age, mean ± SD (range)	68.2 ± 5.0 (50–77)	64.6 ± 4.1 (50–69)	72.2 ± 2.0 (70–77)	<0.001
eHEALS, mean ± SD (range)	24.1 ± 8.6 (8–40)	25.6 ± 9.2 (10–40)	22.4 ± 7.6 (8–40)	0.176
SOTU assessment				
Negative SOTU, mean ± SD (range)	4.0 ± 2.8 (0–9)	3.7 ± 2.8 (0–9)	4.3 ± 2.8 (0–9)	0.382
Neutral SOTU, mean ± SDa (range)	4.5 ± 2.7 (0–9)	4.8 ± 2.7 (0–9)	4.1 ± 2.8 (0–9)	0.310
Positive SOTU, mean ± SD (range)	0.6 ± 1.0 (0–4)	0.5 ± 0.9 (0–4)	0.6 ± 1.1 (0–4)	0.777
Total SOTU, mean ± SD (range)	14.6 ± 3.3 (9–22)	14.8 ± 3.3 (9–22)	14.3 ± 3.3 (9–20)	0.503
Satisfaction with the training, mean ± SD (range)	29.7 ± 7.6 (8–40)	31.2 ± 7.2 (14–40)	27.8 ± 7.8 (8–40)	0.107

**Table 3:** eHEALS value at the baseline (T0) and at the end of the course (T1).

	T0			T1			*p*
	1–2 (%)	3 (%)	4–5 (%)	1–2 (%)	3 (%)	4–5 (%)	
I know how to find helpful health resources on the Internet, n (%)	25.8%	32.8%	41.4%	8.7%	34.5%	56.9%	0.002
2. I know how to use the internet to answer my health questions, n (%)	27.6%	37.9%	34.5%	12.1%	29.3%	58.6%	0.001
3. I know what health resources are available on the Internet, n (%)	26.4%	33.3%	40.3%	17.3%)	29.3%	53.4%	0.004
4. I know where to find helpful health resources on the Internet, n (%)	31.6%	35.1%	33.3%	17.6%	31.6%	50.9%	<0.001
5. I know how to use the health information I find on the Internet to help me, n (%)	31.6%	33.3%	35.1%	19.3%	31.6%	49.1%	0.033
6. I have the skills I need to evaluate the health resources I find on the Internet, n (%)	36.2%	29.3%	34.5%	17.8%	30.4%	51.8%	0.040
7. I call tell high-quality from low-quality health resources on the Internet, n (%)	31%	34.5%	34.5%	17.2%	29.3%	53.4%	0.006
8. I feel confident in using information from the Internet to make health decisions, n (%)	56.1%	22.8%	21%	32.7%	29.3%	37.9%	0.030
eHEALS, mean ± SD	24.3 ± 8.9			28.4 ± 8.1			0.001

1 = completely disagree; 5 = completely agree.

**Table 4:** Money that users would pay for the course (*p* = 0.004).

Cost	n (%)	Satisfaction with the Training
Free	13 (22.8%)	24.8 ± 9.2
1–30 €	3 (5.3%)	21.3 ± 5.0
31–50 €	11 (19.3%)	29.5 ± 3.9
51–70 €	12 (21.1%)	32.1 ± 6.7
71–100 €	11 (19.3%)	35.3 ± 6.4
Over 100 €	7 (12.3%)	31.9 ± 5.3

**Table 5:** Correlation coefficient between eHEALS, SOTU, and satisfaction with training, and their significance.

Domains	ρ	*p*
Negative SOTU	0.24	0.076
Neutral SOTU	−0.16	0.244
Positive SOTU	−0.27	0.048
Total SOTU	−0.29	0.032
Satisfaction with the training	0.78	0.000

## Data Availability

Data supporting reported results can be found in Mendeley Data at https://data.mendeley.com/datasets/c34pt8b48g/1 (accessed on 21 September 2021). DOI 10.17632/c34pt8b48g.1.
